# Sensory Stimulation-Dependent Plasticity in the Cerebellar Cortex of Alert Mice

**DOI:** 10.1371/journal.pone.0036184

**Published:** 2012-04-26

**Authors:** Javier Márquez-Ruiz, Guy Cheron

**Affiliations:** 1 División de Neurociencias, Universidad Pablo de Olavide, Sevilla, Spain; 2 Laboratory of Electrophysiology, Université de Mons, Mons, Belgium; 3 Laboratory of Neurophysiology and Movement Biomechanics CP601, Université Libre de Bruxelles, Brussels, Belgium; Tokyo Medical and Dental University, Japan

## Abstract

*In vitro* studies have supported the occurrence of cerebellar long-term depression (LTD), an interaction between the parallel fibers and Purkinje cells (PCs) that requires the combined activation of the parallel and climbing fibers. To demonstrate the existence of LTD in alert animals, we investigated the plasticity of local field potentials (LFPs) evoked by electrical stimulation of the whisker pad. The recorded LFP showed two major negative waves corresponding to trigeminal (broken into the N2 and N3 components) and cortical responses. PC unitary extracellular recording showed that N2 and N3 occurred concurrently with PC evoked simple spikes, followed by an evoked complex spike. Polarity inversion of the N3 component at the PC level and N3 amplitude reduction after electrical stimulation of the parallel fiber volley applied on the surface of the cerebellum 2 ms earlier strongly suggest that N3 was related to the parallel fiber–PC synapse activity. LFP measurements elicited by single whisker pad stimulus were performed before and after trains of electrical stimuli given at a frequency of 8 Hz for 10 min. We demonstrated that during this later situation, the stimulation of the PC by parallel and climbing fibers was reinforced. After 8-Hz stimulation, we observed long-term modifications (lasting at least 30 min) characterized by a specific decrease of the N3 amplitude accompanied by an increase of the N2 and N3 latency peaks. These plastic modifications indicated the existence of cerebellar LTD in alert animals involving both timing and synaptic modulations. These results corroborate the idea that LTD may underlie basic physiological functions related to calcium-dependent synaptic plasticity in the cerebellum.

## Introduction

The first experimental evidence of long-term depression (LTD) at the parallel fiber (PF)–Purkinje cell (PC) synapse was induced by conjunctive activation of parallel and climbing fiber (CF) inputs in decerebrated rabbit [1] and in slices [2]. This evidence was consistent with the Marr-Albus-Ito models of cerebellar learning [3]–[5]. Subsequently, other types of cerebellar plasticity have been demonstrated [6]. Presynaptic [7] and postsynaptic [8] long-term potentiation (LTP) at the PF–PC synapse supported the concept of bidirectional plasticity controlled by intracellular Ca^2+^ signaling [9], [10]. When CFs were stimulated at 5 Hz, the CF–PC synapses underwent LTD [11].

Exploration studies of the cellular and molecular mechanisms implicated in LTD at the PF–PC synapses have clarified the involvement of the metabotropic glutamate receptor subtype 1 [12], protein kinase C [13], and clathrin-mediated internalization of postsynaptic AMPA receptors [14]. Other studies have shown that the activation of the postsynaptic *α*-calcium/calmodulin-dependent protein kinase II and the nitric oxide–cyclic GMP-protein kinase G cascade [15] were also involved in LTD. In addition, LTD was associated with both presynaptic [16] and postsynaptic [17] NMDA receptors.

PF–PC LTD and LTP have been confirmed in decerebrate animals [18], [19]. LTD and LTP were also shown to occur in the granular cell layer following facial tactile stimulation in the anesthetized rat [20]. That study further demonstrated that changes in local field potential (LFP) amplitudes were accompanied by changes in latency, which indicated a possible role of plasticity in the spatio-temporal spike sequences of the cerebellar input. Of interest, Wang et al. [21] demonstrated a PF–PC LTP following high-frequency stimulation (100 Hz) in the PF of anesthetized mice. However, to date, no work had demonstrated either LTP or LTD in alert animals.

Here, we investigated the existence of LTD plasticity in alert mice by using electrical stimulation of the whisker pad. We found that LTD occurred in the N3 LFP component (amplitude decrease and delayed latency), after 10 min of whisker stimulation at a frequency of 8 Hz. The LFP N2–N3 component coincided with the early evoked simple spikes (SS) of the PC and was followed by an evoked complex spike (CS); this conjugated activation of the PC was reinforced during the 8-Hz stimulation. Our results provide the first demonstration that LTD can be induced in the cerebellum of alert animals in response to sensory peripheral input.

## Materials and Methods

### Animals

Male FVB mice (n = 30), ages 5–8 months, obtained from an authorized supplier (Charles River Laboratories, Wilmington, MA, USA), were used as experimental animals. All animal procedures were approved by the University of Mons Ethics Committee and conducted in conformity with the European Union directive 609/86/EU. Every effort was made to minimize the number of animals and their discomfort.

### Surgical preparation

Animals were prepared for chronic recordings of LFP and PC single-unit activity [22]. Mice were anesthetized with xylido-dihydrothiazin (Rompun©, Bayer, 10 mg/kg) and ketamine (Ketalar©, Pfizer, 100 mg/kg). Animals were administered an additional dose of xylido-dihydrothiazin (3 mg/kg) and ketamine (30 mg/kg) when they demonstrated agitation or marked increases in respiration or heart rate during the procedure. In addition, local anesthesia (0.5 mL of 20 mg/mL lidocaine and adrenaline [1∶80000, Xylocaine©, Astra Zeneca]) was administered subcutaneously during the soft tissue removal. During surgery, two small bolts were cemented perpendicular to the skull to immobilize the head during the recording sessions, and a silver reference electrode was placed on the surface of the parietal cortex. To allow access to the Crus I and II areas in the cerebellum, an acrylic recording chamber was constructed around a posterior craniotomy (2×2 mm) and covered with a thin layer of bone wax (Ethicon©, Johnson & Johnson).

### Stimulation of the whisker region

**Figure 1:**
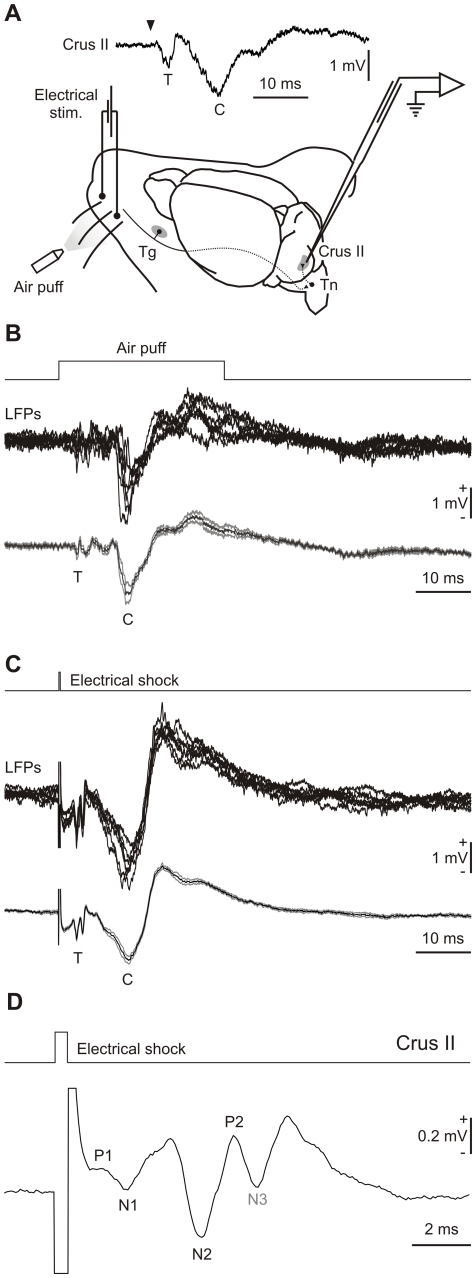
Experimental design and electrophysiological response to electrical stimulation of mouse whiskers. (***A***) Animals were prepared for chronic recordings of local field potentials and unitary extracellular activity in the Purkinje cell layer of the Crus I/II area. Facial dermatomes of the whisker region were electrically or tactilely stimulated with a pair of needles under the skin (Stim.) or an air puff pulse, respectively. Sensory information comes into the Crus I/II area from the trigeminal nucleus (Tn) in the brainstem, which receives afferent signals from the trigeminal ganglion (Tg). The LFP recorded in the alert animal induced by tactile stimuli consisted of two major negative waves corresponding to trigeminal (T) and cortical (C) responses (upper trace). (***B***) In some of the recordings, the T component appeared as two separate components (N2 and N3). The figure shows superimposed LFPs (upper trace, n = 7) and the corresponding mean average (with error bars in gray at bottom) for the LFP acquired after tactile stimulation of the whisker. (***C***) Superimposed LFPs (upper trace, n = 7) and the corresponding mean average (with error bars in gray at bottom) for the LFP acquired at the same recording place shown in *B* but after electrical stimulation of the whisker. A major reproducibility of the T-related components in the superimposed traces and a decrement in the error bars in the mean average of LFP were observed after electrical stimulation in comparison to air puff stimulation. (***D***) The lower trace shows T-related components enlarged from a representative local field potential recording from the Crus II area in the Purkinje cell layer in response to a single-pulse electrical stimulation of the whisker pad (Stim. trace). Early response associated with sensory input in the cerebellum via the trigeminal nucleus is characterized by P1-N1-N2-P2-N3 components.

Facial dermatomes of the whisker regions were stimulated by air puffs or electrical pulses. Whisker regions were electrically stimulated with a pair of small cutaneous needles inserted under the skin (inter-electrode distance 3–4 mm; Fig. 1*A*). Electrical stimulation consisted of a single square pulse, 0.2 ms in duration and <2 mA current intensity, delivered by an isolation unit (Isoflex, AMPI, Israel) connected to an analog pulse generator (Master 8, AMPI, Israel). The amplitude of the current was adjusted to avoid overt movements and animal discomfort. LFPs close to the PC layer and PC single-unit activity were recorded in response to single electrical stimuli delivered at semi-random intervals of 10±3 s in the whisker region. When tactile stimulation was required, air puffs were applied at the same rate to the whiskers (2.6 bar and 30 ms in duration; Fig. 1*A*) by an air pressure system (Picospritzer).

### Paradigm used for inducing LFP plasticity

Preliminary experiments were performed to test the direct effect of different frequency trains of stimulation on the LFPs. For this, 30 electrical stimuli were delivered at different frequencies between 0.2 and 50 Hz. After the analysis of the direct effects on the latencies and amplitude of the different LFP component, the 8-Hz stimulation frequency was selected and used for inducing plasticity. An experimental session consisted of a 15-min control situation in which single electrical stimuli were given at intervals of 10±3 s in the whisker region. This period was immediately followed by 10 min of 8-Hz stimulation and then by 30 min of control during which the same single stimuli were applied at the same frequency as in the control situation. Additional controls were performed in different animals in which the period of 10 min at 8 Hz was replaced by 10 min at the same frequency as the control situation.

### Single-unit and multiple-unit recordings in alert mice

Twenty-four hours after anesthesia, alert mice were restrained for the recording session. The dura was removed over the cerebellum to expose the tissue in the recording chamber. Recordings were performed in the Crus I or II area, and the depths of the electrodes were noted. To avoid unnecessary stress for the animals and movement artifacts, recording sessions were performed in a quiet room when animals were awake and calm. Single and multiple recordings were performed with seven, linearly arranged, quartz-insulated, platinum-tungsten fiber microelectrodes (outer and shaft diameters of 80 µm and 25 µm, respectively), with 250-µm inter-electrode spacing (Thomas Recordings©). Each microelectrode was mounted into a stretched elastic rubber tube to enable proper positioning via DC-micromotors (resolution of 0.27 µm). Neural activity signal recordings were filtered at 100 Hz high-pass and 10 kHz low-pass. LFP and unitary electrical activities were stored digitally on a computer after conversion with an analog–digital converter (Power 1401, CED©, Cambridge, UK). The recorded data were digitized continuously at 20 kHz. Off-line analysis and illustrations were performed with Spike2 CED software (CED©, Cambridge, UK).

A neural signal was considered to originate from a PC when it presented two types of spiking activities: SS, characterized by a single depolarization (300–800 µs) that occurred between 20 and 200 Hz; and CS, characterized by an initial fast depolarization (300–600 µs), followed by smaller and relatively constant wavelets. Simple and complex spikes were considered to originate from the same PC when a transient pause (∼15 ms) in SS firing followed each CS. Recordings were analyzed when a stable signal was present for longer than 60 s. Electrophysiological responses to electrical stimulation in the whisker region were assessed by both the configuration of the LFP (which must show P1-N1-N2-P2-N3 components, Fig. 1*D*) and by the identification of appropriate PC firing (modulated by spontaneous whisker movements and electrical stimulation). The P1/N1 component was taken to reflect the evoked activity in the mossy fibers of the granule cell (GC) layer. When this component was stable during the experiment, we assumed there was no extracerebellar modification of the input signals.

### Collision testing between the whisker pad stimulation and direct PF stimulation

Stimulation of the PFs was delivered by a bipolar parylene-coated microelectrode placed just below the surface of the cerebellar cortex [21] 0.5 mm laterally from the recording electrode. The position of the bipolar electrode was adjusted to produce a negative field in the superficial part of the molecular layer corresponding to the activity of the PF–PC synapses. Then the presence of stable LFP triggered by the whisker pad stimulation was checked before the application of a single electrical stimulus at the PF. To produce a collision between the afferent volley traveling in the pathway between the GC and the PC, and the antidromic volley elicited by the PF stimulation, the latter was applied 2 ms before the whisker pad stimulation. The amplitude of each LFP component was then compared before, during, and after the collision test.

### Data analysis

The peak-amplitudes and latencies of N2 and N3 components were used as an index of the postsynaptic responses [21], [23], [24]. These were compared with the SS responses of the recruited PCs. Data were analyzed off-line for amplitude and latency quantifications of N1, N2, and N3. For this analysis, 30 successive evoked field potentials were averaged to obtain one average data point every 5 min. The amplitude was computed by peak-to-peak measurements. For this calculation, negative peaks were compared to the trough of the preceding positive wave. When the P1 positive peak was not evident, we used the inflection point observed in the averaged trace for the N1 measurement. For N3 measurements, the amplitude was set as the difference between the positive peak between N2 and N3 and the negative peak of N3. Latency was determined as the time difference between stimulus onset and N1, N2, or N3 averaged peaks. For comparisons between animals, amplitude values for each one of the components of the LFP were normalized for statistical analysis.

Discrimination between PC simple and complex spikes was performed with Spike2 CED software (CED, Cambridge, UK) and controlled visually before analysis. Waveform averaging was performed with the corresponding function of this software on a 120-s minimum recording. The SS firing response was evaluated on an averaged histogram that included all the stimulations during the recording of a given cell (bin size = 1 ms). The significance of the response was evaluated by comparing the averaged spike number per bin on five successive bins following stimulation to the bin values in the interval (−200 to −100 ms) before stimulation. The effect of a single stimulation on PC firing was also analyzed by superimposing single trace trials, which allowed the identification of evoked simple and complex spikes. A CS firing response was defined as the occurrence of a CS during the interval (10–50 ms) following at least 30% of the stimulations [25].

The student's *t* test and one-way ANOVA for repeated measures were performed in SPSS (v 14.0, SPSS Inc©). Statistical significance was set at p<0.05. Unless indicated otherwise, the results are shown as mean±SEM.

## Results

The electrophysiological response to electrical and tactile whisker stimulation near the PC layer of the Crus I or Crus II area (at 10±3 s intervals) was characterized in the alert mice. The LFP recorded in the alert animal induced by tactile stimuli consisted of two major negative waves (Fig. 1*A*). These two components have been previously reported in anesthetized rats as T and C, corresponding to trigeminal and cortical responses [20], [26], [27]. In the present condition, T and C components peaked at 4.5±3.5 ms and 16.1±9.4 ms (mean±SD; n = 5), respectively. In some of the performed recordings, the T component appeared as two separated components (Fig. 1*B*). In these instances, the latency of the two peaks was 2.6±0.5 ms and 3.8±0.5 ms and 12.4±3.4 ms for the C component (mean±SD; n = 5).

To reduce the variability of the LFP observed after tactile stimulation by an air-puff pulse in alert mice, we tested subcutaneous electrical stimulation of whiskers. This alternative sensory stimulation protocol allows a diminishing of the duration of the stimulus (0.2 ms vs 30 ms for air pulse), increasing the reproducibility of the responses. Figure 1 shows the superimposed LFPs (n = 7) and the corresponding mean average (with error bars in gray) for the LFP acquired at the same recording location after tactile (*B*) and electrical stimulation of the whisker (*C*). A major reproducibility of the T-related components in the superimposed traces and a decrement in the error bars in the mean average of LFP were observed after electrical stimulation in comparison to air puff stimulation. In consequence, electrical stimulation of whiskers was used to characterize plastic changes in the cerebellum of the alert mice.

### Presynaptic and postsynaptic components of the LFP

LFP recorded near the PC layer after electrical stimulation of the whisker comprised three well-defined waves appearing before the slower C component (Fig. 1*D*). In this experiment, only T-related components were analyzed because of their direct relation with sensory input in the cerebellum via the trigeminal nucleus [27]. These three early negative waves, designated here as N1, N2, and N3, peaked at 1.8±0.4 ms, 3.3±0.3 ms, and 4.5±0.2 ms (mean±SD; n = 15), respectively, after stimulus onset. The mean peak-to-peak amplitude of these components corresponded to 0.19±0.15 mV, 0.43±0.18 mV, and 0.48±0.19 mV, respectively (mean±SD; n = 15). The short latency and small amplitude of N1 suggested a correspondence with the classical P1–N1 presynaptic input component [23] originating in the mossy fiber in the GC layer, originally documented following juxtafastigial stimulation in anesthetized cats. Additional experiments were required to clarify the nature of the later N2 and N3 components. To this effect, we attempted different approaches, namely (1) PC unitary extracellular recording, (2) depth profile analysis of the induced field, and (3) collision testing between whisker stimulation and direct PF stimulation.

**Figure 2:**
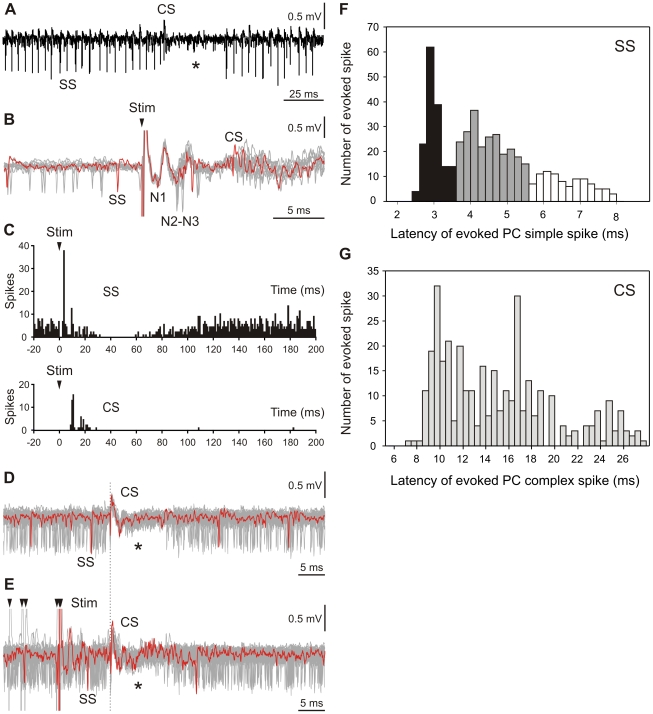
Relationships between evoked local field potential components and Purkinje cell firing behavior. (***A***) Recording of spontaneous firing behavior of a Purkinje cell (PC) shows the presence of single spikes (SS) and complex spikes (CS). The presence of a CS followed by a pause in the SS firing (asterisk) identifies this neuron as a PC. (***B***) Single trials, superimposed (n = 11), show spontaneous firing before the whisker electrical stimulation (Stim) and the temporal reorganization of the firing after the stimulus. SS firing occurred at the low points of the N2 and N3 components and later. The evoked CS occurred at a latency of 9–13 ms after the stimulus onset (arrowhead). The single trace appears in color to facilitate the identification of SS and CS. (***C***) Histogram (bin size = 1 ms) of a PC recording shows the typical SS (top) and CS (bottom) response to whisker pad electrical stimulation (n = 37). After stimulus onset (arrowhead), the PC showed an initial SS burst at N2–N3 latencies, followed by a CS, followed by a silent period. (***D***) Superimposition (n = 43) of a PC recording triggered by CS (dotted line) during spontaneous firing. (***E***) Superimposition (n = 58) of the same PC presented in (***C***) in response to electrical whisker stimulation (arrowheads). The figure shows that SS and CS waveforms were preserved during whisker stimulation. (***F***) The SS latency distribution, in response to whisker pad electrical stimulation, combined from 45 PCs. The two major peaks correspond to the occurrence times of the N2 (black bars) and N3 (gray bars) postsynaptic components. Bin sizes are 0.2 ms. (***G***) Latency of the complex spike (CS) for the same population of PCs. The CS always occurred after SS evoked potentials. Bin sizes are 0.5 ms.

To characterize the relationship between the potentially postsynaptic components of the LFP recorded near the PC layer and the firing behavior of PCs, single electrical stimuli were applied to the whisker pad (at 10±3 s intervals) during extracellular unitary recording of the identified PC. A total of 45 identified PCs were included in the analysis. A neural signal was considered to originate from a PC when it presented two types of spiking activities: SS, characterized by a single depolarization (300–800 µs) that occurred between 20 and 200 Hz; and CS, characterized by an initial fast depolarization (300–600 µs), followed by smaller and relatively constant wavelets. Simple and complex spikes were considered to originate from the same PC when a transient pause (∼15 ms) in SS firing followed each CS (Fig. 2*A,D,E* asterisks). PC response to electrical whisker stimulation consisted of SS and CS generation. In Figure 2*B*, superimposition of single trials (n = 16) showed spontaneous SS firing before the stimuli and SS and CS evoked by electrical stimulation of whiskers (Stim in Fig. 2*B*). Of interest, SS occurred with the same latencies as the above-mentioned N2 and N3 components (3.5–4.5 ms, in this case; Fig. 2*B*) whereas the CS occurred 9–13 ms after the stimulation. For the majority of the recorded PCs, a very short silent period of 3–4 ms occurred after the early SS response and preceded the arrival of the CS.

The histogram (Fig. 2*C*, top) shows the initial SS increase followed by a CS increase (Fig. 2*C*, bottom); this finding confirmed the consistent temporal relationship between the peripheral stimulation and these early SS responses. A later inhibition occurred between 24 and 100 ms after the stimulus. In Figure 2, superimposition of the same PC recording triggered by CS (dotted line) is shown during spontaneous firing (n = 43, Fig. 2*D*) or in response to electrical whisker stimulation (n = 58, arrowheads in Fig. 2*E*). One of the traces is shown in red to facilitate identification of the SS and CS. This approach, applied to each of the recorded cells, allowed us to confirm that the spontaneous CS originated from the same PC as the CS evoked by stimulation. Therefore, the PC response was characterized by a short excitation (SS+CS) followed by a longer inhibition, with a variable duration (20 ms to 150 ms). In the analyzed PC population (n = 45), the onset of the first evoked spike occurred at a mean latency of 3.0±0.5 ms (Fig. 2*F*, black bars) whereas the onset of the second group of spikes had a mean latency of 5.2±1.2 ms (mean±SD; measured in 45 PCs) (Fig. 2*G*, gray bars). The first two peaks observed in the distribution of SS latencies measured in the PC population corresponded to the time of the second and third LFP components, respectively. For the same PC population, the CS onset always occurred after the evoked SS, at a mean latency of 14.8±5.1 ms (Fig. 2*F*). Therefore, results from PC spikes recording after whisker electrical stimulation strongly suggest that N2 and N3 waves are associated with postsynaptic components. Although SS activity from PCs was closely associated with the N2 and N3 components, the contribution of GC and Golgi cell placed a few micrometers beyond the recording site must not be discarded.

**Figure 3:**
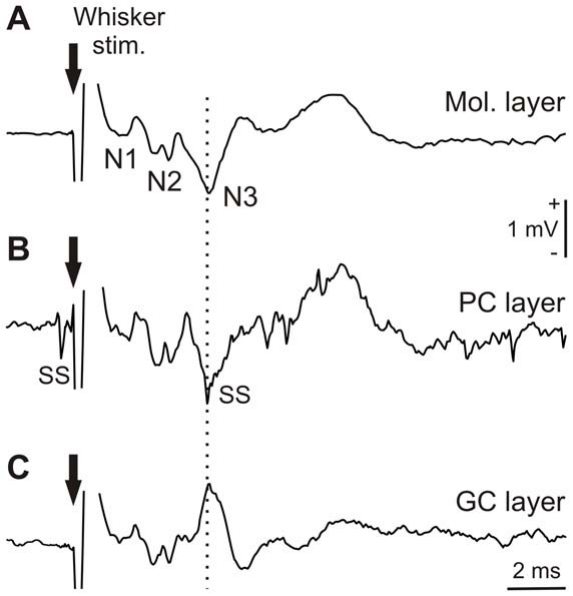
Depth profile analysis of the LFP induced by electrical stimulation. (***A***) LFP recorded in the molecular layer (Mol. Layer). N1, N2, and N3 components are indicated. (***B***) LFP recorded near the PC layer where simple spikes (SS) were recorded. At this level, indexed by the occurrence of PC firing, the N3 amplitude decreased. (***C***) LFP recorded just beneath the PC layer showing the polarity inversion of the N3. Black arrow indicates electrical whisker stimulation. Vertical dotted line indicates N3 latency along different depths.

Furthermore, additional in-depth penetrations were performed to better identify the postsynaptic nature of the N2 and N3 components. When the microfiber penetrated the molecular layer perpendicularly, the negative polarity of the postsynaptic component was maintained until the PC layer was approached (Fig. 3*A*). At this level, indexed by the occurrence of PC firing, the N3 amplitude decreased (Fig. 3*B*). Then, continuing to move in depth, just beneath the PC layer, the postsynaptic field suddenly was changed into a positive component peaking at the exact latency as the previous negative peak (Fig. 3*C*). When such an inversion occurred, it could be repeated and reversed again by moving the microfiber up and down in the same configuration. Of note, we have observed such an inversion only for the N3 component and not for the N1 and N2 components. This pattern indicates that the postsynaptic generator of N3 is produced by PC *dipoles* vertically oriented with the negative pole situated in the superficial part of the dendrite arborization [28]. The nature of N2 is more complex and could correspond to the granular and Golgi cell activity [23] directly followed by the action of an ascending axon–PC synapse [29].

**Figure 4:**
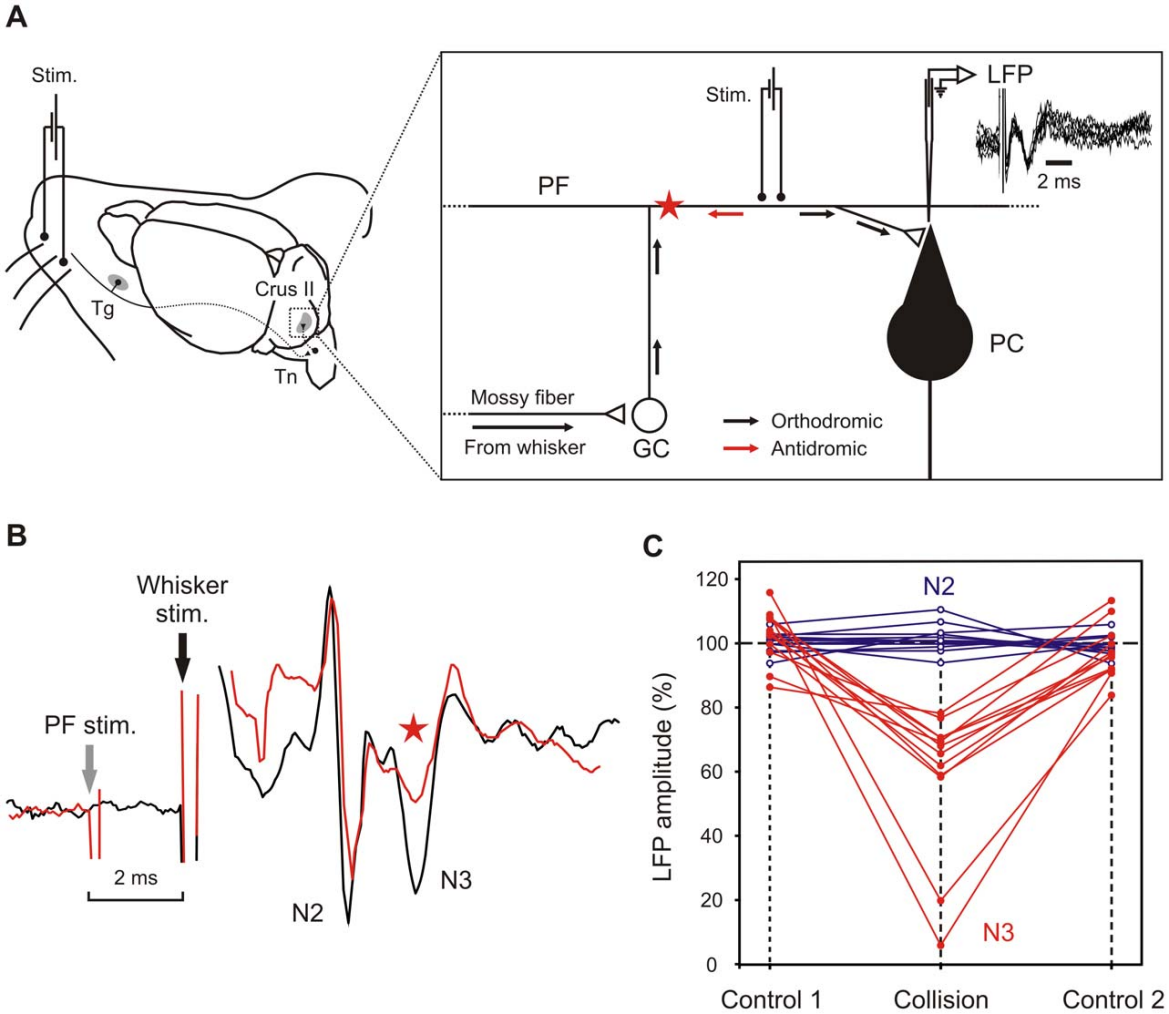
Experimental design and electrophysiological responses during the collision testing. *(* ***A*** *)* Diagram of the neural pathways concerned in the direct stimulation (Stim.) applied on the top of the parallel fiber (PF). This stimulation produced a negative LFP (superimposed traces on the right corner) recorded by a microfiber placed in the dendritic tress of Purkinje cells (PC). The small arrows indicate the propagation of the orthodromic action potentials (black arrows) and the antidromic action potentials (red arrows) producing collision (red star). The peripheral input coming from the whisker pad is transmitted to the granule cells (GC) via the mossy fiber. (***B***) Superimposition of the average LFP (n = 10 stimulations) in control (black trace) and during the collision (red trace, red star). The gray vertical arrow indicates the direct stimulation of the PFs while the black vertical arrow indicates the peripheral stimulation of the whisker pad. (***C***) Evolution of the N2 (blue lines) and N3 (red lines) amplitudes during the collision testing with respect to the control situation recorded before (control 1) and after the collision (control 2).

Finally, to check for the postsynaptic origin of the N2 and N3 components, we electrically stimulated the PF to partly block the afferent volley (elicited by the whisker pad stimulation) traveling to the PC via the ascending axons of the granule cells and the PF beam (Fig. 4*A*). Prior to the collision testing, we first demonstrated that the PF stimulation could induce a postsynaptic LFP at the PC level. This stimulation produced a negative field in the molecular layer, peaking at a latency of about 2.5±0.4 ms (mean±SD; n = 10) for a stimulation distance of about 0.5 mm (superimposed traces in Fig. 4*A*). Then a collision test between whisker pad and the PF stimulation given 2 ms earlier was performed in different animals (n = 5) (Fig. 4*B*). This test induced a significant decrease in the N3 component while N2 and N1 remained unchanged (Fig. 4*C*). N3 decrement could be produced by PF–PC synapse blockade or by collision along the PF while N2 associated with the ascending axon–PC synapse, and the granular and Golgi cell activity remain intact.

### 8-Hz stimulation of the whisker pad had maximal effects on postsynaptic evoked components

**Figure 5:**
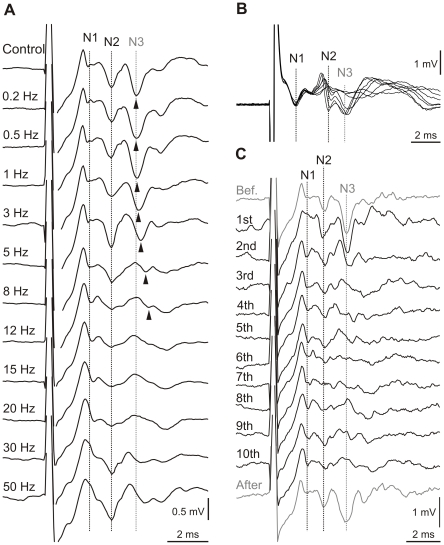
Effects of stimulation frequency on evoked components. (***A***) Representative responses of one mouse to various stimulus trains (30 stimuli per train) at frequencies between 0.2 and 50 Hz (indicated at left). Each trace shows the mean local field potential (averaged over the last 20 stimuli) recorded near the Purkinje cell layer of the Crus I/II area. In control conditions, the stimuli were delivered every 10±3 s. To facilitate visual comparison, dotted lines have been placed to indicate the locations of the N1, N2, and N3 peaks observed before stimulation. The arrowheads indicate shifts in the peak latencies. (***B***) Superimposed traces of mean averaged local field potentials obtained at stimulation frequencies from control to 8 Hz. Traces were aligned with the stable N1 component. Dotted lines indicate the locations of the N1, N2, and N3 peaks observed before stimulation. (***C***) Single-trial traces that correspond to the local field potentials in response to the first 10 stimuli (8 Hz) in a train (numbered at left). Single control stimuli responses were recorded just before the train (top) and just after (bottom) the 10^th^ stimulus (8 Hz).

Different stimulation frequencies induced different plastic changes in the cerebellum [30]; therefore, we tested the immediate effects of whisker stimulation at different frequencies on the N2 and N3 components. On a separate group of mice, involved only in this testing (n = 10), we applied various stimulus trains (30 stimuli per train) with frequencies between 0.2 and 50 Hz. Amplitudes and latencies of the postsynaptic components were obtained by averaging responses to the last 20 stimuli of each frequency train. Figure 5*A* shows the representative average for each stimulation frequency with the changes observed in the N2 and N3 components in one alert animal. The amplitudes of N2 and N3 decreased with increasing stimulation frequencies, reaching minimum values at frequencies between 8 and 20 Hz. N3 showed an increase in the peak latency (arrowheads in Fig. 5*A*). In contrast, N1, which reflected the presynaptic input that originated from the mossy fiber in the granule cell layer, showed stable amplitude and latency with stimulation trains between 0.2 and 8 Hz; this result indicated that there were no significant extracerebellar modifications of the input signals. These points are illustrated by the superimposition of the average LFPs from different frequency stimulations in a different animal (ranging from the control situation to 8 Hz; Fig. 5*B*). The effects on N2 and N3 appeared early in the stimulation train, but they were not sustained beyond the short-duration train (Fig. 5*C*, after); thus, they did not correspond to plastic changes: i.e., after the 30^th^ stimulus in the train, a control stimulation induced N2 and N3 peaks similar to those observed in the first stimulus.

**Figure 6:**
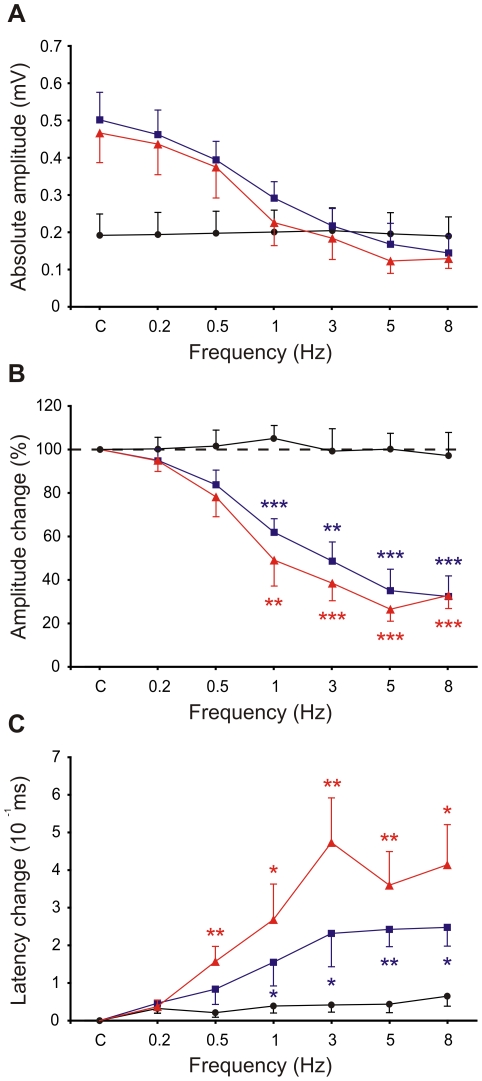
Changes in amplitude and latency of evoked local field potential components along the whisker stimulation frequency range. (***A***) Absolute amplitude of postsynaptic components at stimulations of 0.2, 0.5, 1, 3, 5, and 8 Hz. Changes in mean amplitudes (***B***) and latencies (***C***) compared to control conditions were induced by stimulation frequencies of 0.2, 0.5, 1, 3, 5, and 8 Hz. N1 (black circles), N2 (blue squares), and N3 (red triangles) components are expressed as mean±SEM. Significant differences (asterisks) were evaluated for the postsynaptic components (N2 and N3) with respect to the presynaptic input (N1). (p<0.001, ***; p<0.01, **; p<0.05, *).

To quantify the effects of frequency stimulation on postsynaptic components, absolute amplitude and changes in amplitude and latency were assessed for stimulations of 0.2, 0.5, 1, 3, 5, and 8 Hz in 10 animals. In this frequency range, N1 remained stable (Fig. 6*A*). We therefore used the N1 amplitude as a reference for studying changes in other components. The changes in N2 and N3 amplitudes were significantly different at 1, 3, 5, and 8 Hz compared to N1 (p<0.05; n = 10) (Fig. 6*B*). However, N2 and N3 showed similar changes in amplitude. Latency changes were calculated as the difference between the peak time under control conditions and at each stimulation frequency. N2 and N3 latency changes increased with increasing frequencies; at 0.5–8 Hz and at 1–8 Hz (p<0.05; n = 10), respectively, they were significantly different from the N1 latencies (Fig. 6*C*). No significant differences in latency were observed between N2 and N3. In the remaining analyses, we adopted the 8-Hz stimulation protocol for inducing plasticity.

### Whisker stimulation at 8 Hz induced LTD of postsynaptic components

**Figure 7:**
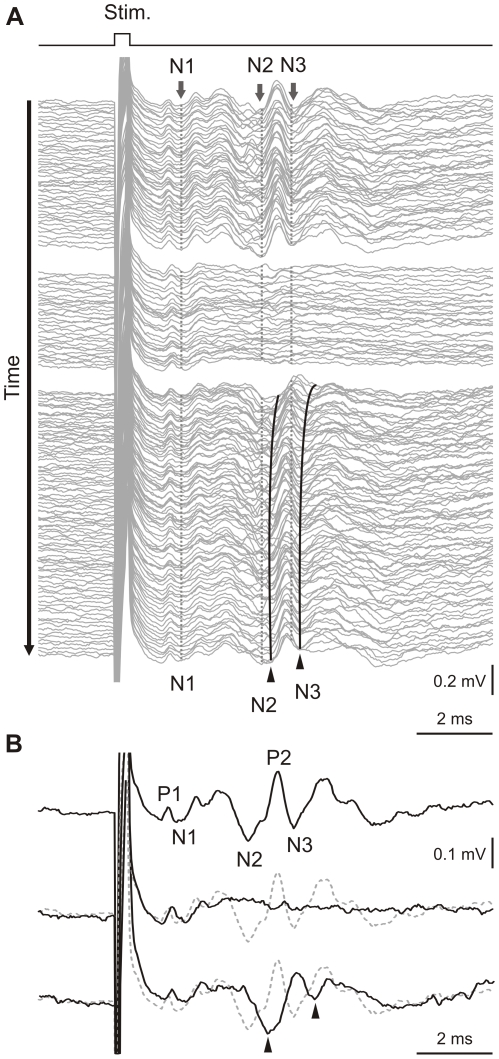
Long-term depression evoked by 8-Hz electrical stimulation of mouse whiskers. (***A***) Continuous recordings arranged in cascades show single traces of evoked local field potential components induced by whisker electrical stimulation. (Top group) 15 min in control conditions; (middle group) 10 min of 8-Hz stimulation; (bottom group) 30 min after the stimulation protocol. Vertical dotted lines indicate the peak latencies of N1 presynaptic and N2 and N3 postsynaptic components under control conditions. During the 8-Hz stimulation, the postsynaptic components almost disappeared, and the presynaptic input remained stable. Long-term depression is evident after the 8-Hz stimulation protocol (bottom group), when the latency of the N2 components (curved black lines next to dotted lines) increased and the amplitude of the N3 component strongly decreased. These effects were maximal just after the 8-Hz stimulation and persisted for at least 30 min. (***B***) Single traces selected from (***A***) to compare latencies and amplitudes of evoked field potential components; (top) before 8-Hz stimulation protocol, (middle) during 8-Hz stimulation, and (bottom) just after 8-Hz stimulation protocol. Arrowheads indicate shifted latencies of postsynaptic components after 8-Hz stimulation. Dashed lines indicate the locations of components in the control condition to facilitate comparison. Calibrations are indicated for each figure.

The LTD protocol comprised an 8-Hz electric stimulation of the whisker pad delivered over 10 min in the alert animal. To explore eventual changes in the presynaptic and postsynaptic components, we continuously recorded the LFPs induced by single electrical stimulations (every 10±3 s) before (15 min) and after (30 min) the protocol. Figure 7 shows the evolution of LFPs recorded near the PC layer of the Crus II area in response to electrical whisker stimulation in one alert mouse. The traces in Figure 7*A* represent single-trial LFPs induced by single electrical stimulations before (15 min, Fig. 7*A*, top), during (10 min, 8 Hz; Fig. 7*A*, middle), and immediately after the 8-Hz stimulation protocol (30 min, Fig. 7*A*, bottom). After the 10-min 8-Hz stimulation, N2 and N3 peak latencies increased compared to control conditions. In addition, a marked decrease was observed in the N3 amplitude (Fig. 7*B*).

**Figure 8:**
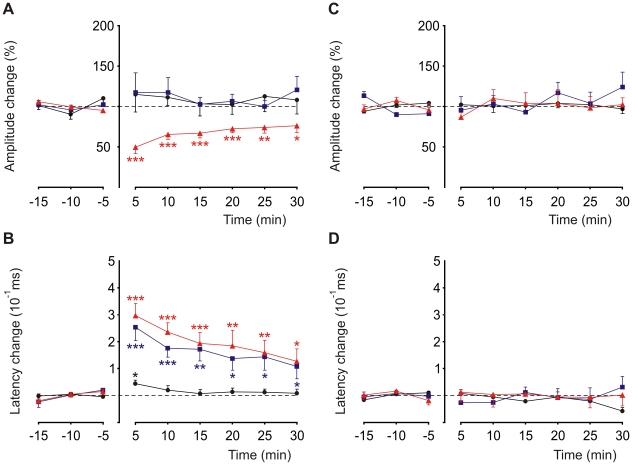
Time course of the effects induced by an 8-Hz stimulation train on evoked field potential components. (***A***) Time course of amplitude changes for N1 (black circles), N2 (blue squares), and N3 (red triangles) before (negative time periods) and after (positive time periods) the 8-Hz stimulation protocol (not shown). Mean normalized values were calculated for each 5-min interval of data from 15 alert animals. Data points are mean±SEM. (***B***) Time course of N1 (black circles), N2 (blue squares), and N3 (red triangles) latency changes before (negative time periods) and after (positive time periods) the 8-Hz stimulation protocol (not shown). Mean data represent the time difference at peak latency between each 5-min interval and the mean value measured in control conditions (negative time periods). Significant differences from control are indicated with asterisks. A small but significant (*) difference observed in the N1 presynaptic component showed recovery to control values after the first 5 min. (***C***, ***D***) Controls were performed in different animals (n = 10) for which the 10-min period at 8 Hz was replaced by 10 min at the same frequency as the control situation. No significant changes were observed in the amplitude or latency compared to the control condition. (p<0.001, ***; p<0.01, **; p<0.05, *). Data points are mean±SEM.

Mean LFP changes were quantified in 15 animals by comparing averages over each 5-min interval before and after 8-Hz stimulation (Fig. 8*A, B*). The N3 amplitude was significantly decreased compared to the control condition (p<0.05; n = 15). This effect was maintained during the 30 min of recordings, with a maximum (∼50%) observed just after the 8-Hz stimulation trains (Fig. 8*A*, red triangles). In contrast, no significant changes in amplitudes were observed for the N1 or N2 components (Fig. 8*A*, black circles and blue squares, respectively). N2 and N3 showed significant latency increases compared to control (p<0.05; n = 15). These latency changes were maintained during the 30 min of recordings, with a maximum (∼300 µs) observed just after the 8-Hz stimulation trains (Fig. 8*B*, red triangles and blue squares). The N1 latency showed a small but significant change just after 8-Hz stimulation (p<0.05; n = 15); however, in contrast to N2 and N3, the N1 latency recovered to control values after the first 5 min of recording (Fig. 8*B*, black circles). Controls were performed in different animals (n = 10) for which the 10-min period at 8 Hz was replaced by 10 min at the same frequency as the control situation. No significant changes were observed in the amplitude (Fig. 8*C*) or latency (Fig. 8*D*) compared to the control condition. To verify the specificity of the reported LTD effects, we used 0.5-Hz stimulation in place of the 8-Hz stimulation protocol. In this case, the N3 LTD effects on both latency and amplitude were absent.

### SS and CS during the 8-Hz stimulation protocol

**Figure 9:**
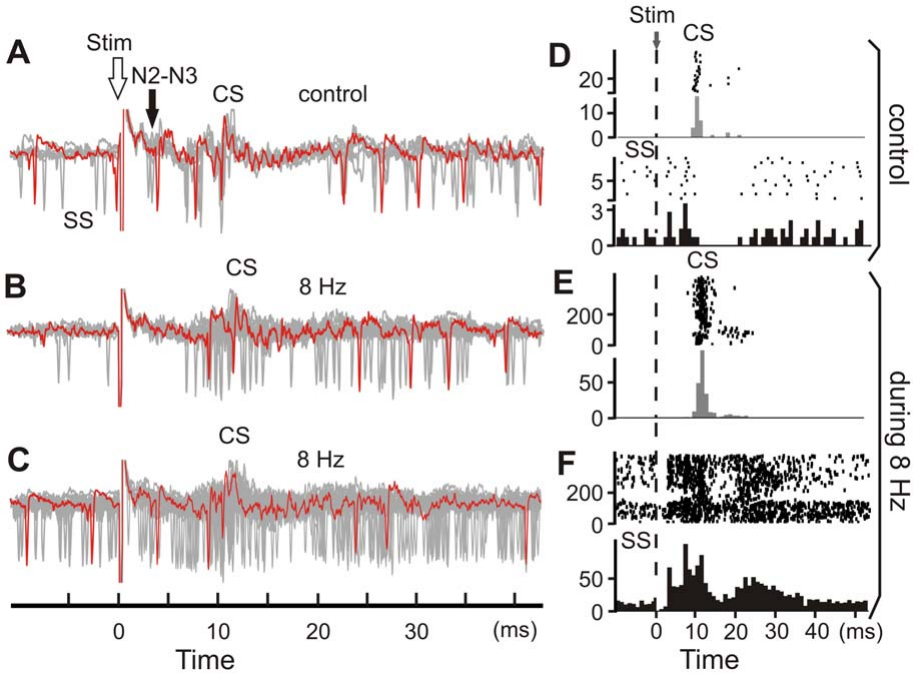
Simple and complex spike firing behavior before and during the 8-Hz electrical stimulus train. (***A***) Superimposed single stimulus (Stim, open arrow) trials under control conditions, before the 8-Hz stimulation. Note the SS evoked responses at the latency of the N2–N3 local field potential components (N2–N3, filled arrow). (***B***, ***C***) Superimposed single traces during two 8-Hz stimulation protocols. In each case (***A***–***C***), the stimulations (Stim, open arrow) are aligned for comparisons. Single trace appears in color to facilitate the identification of SS and CS. Of note, (***B***) the early evoked SS is absent, and (***C***) the evoked SS is desynchronized compared to the control situation (***A***). (***D***) Raster sweeps (top) and related histograms (bin size = 1 ms, bottom) of the CS (upper part) and SS (lower part) evoked responses in control conditions. (***E***, ***F***) Raster sweeps (top panels) and histograms (bin size = 1 ms, bottom panels) of (***E***) the CS and (***F***) SS evoked responses during the 8-Hz stimulation.

To identify more precisely the PC output signals during the 8-Hz stimulation protocol, we analyzed PC recordings (n = 10 PCs in 5 mice) in which both SS and CS responses remained visible, despite the high-frequency stimulation (Fig. 9). We found that the SS frequency doubled from 38.2±30.0 Hz before the 8-Hz stimulation to 73.7±29.3 Hz during the stimulation (p<0.0001). The evoked CS frequency increased from 0.8±0.3 Hz before the 8-Hz stimulation to 5.2±1.5 Hz during the stimulation (p<0.0001). Moreover, during the 8-Hz stimulation, the CS occurred at a mean latency of 20.4±6.5 ms.

## Discussion

This study demonstrated the existence of an LTD in the cerebellar cortex of alert mice. This LTD was characterized by a specific decrease in the N3 amplitude accompanied by an increase in the latency peaks of the N2 and N3 LFP components.

### Origins of the N2 and N3

The origin of the LFP components exhibiting the reported plasticity can be approached by the in-depth recording showing a polarity inversion of N3 at the PC level. This result may indicate that this component is produced by superficial dendrites of the PC depolarized by extracellular current flow from the passive sources in the deeper dendrites and PC soma (dipoles vertically oriented, negativity up/positivity down) as proposed by Eccles et al. [31]. Indeed, this specific inversion, demonstrated for the first time in alert animals following peripheral stimulation, was previously demonstrated with electrical stimulation of the juxtafastigial region in the anesthetized cat (see Fig. 10 I in Eccles et al. [23]) and during cutaneous electrical stimulation of the snout in decerebrate and pentobarbitone-anesthetized rats [32]. Moreover, the physiological origin of the present N3 is reinforced by the collision test demonstrating a specific decrease of the N3 component when the PFs were directly stimulated just before (∼2.0 ms) the whisker pad stimulation. Similarly, the N3 evoked by the juxtafastigial stimulation was also suppressed by a concomitant PF stimulation in the cat [23]. This result suggests that the sensory input contributing to the N3 travels along the PF beam and that N3 reflects the activity of PF–PC synapses. We note here that although the assumption that PF stimulation effects are caused by collision along the pathway from the GC to the PC via the PF, additional effects induced by other elements of the cerebellar cortex cannot be ruled out. Nevertheless, the collision test finding was also reinforced by the timing of the SS synchronized on the N3 field. A similar conjunction has already been illustrated in Figure 9*A* in Eccles et al. [23]. At the same time, the fact that N2 is not modified by either the collision or depth inversion points to a different site of generation. N2 could possibly reflect the conjoint activity from the granule and Golgi cells, corresponding to the N2 component recorded *in vitro* and in anesthetized preparation [20], [30], and/or the synapse between the ascending segment of the GC and PC at the soma level.

Although our results have established that the evoked SS and the N2 and N3 components occurred simultaneously, the exact nature of the involved excitatory synapses remains to be clarified. Two non-exclusive possibilities can be considered. First, the early evoked SS may arise from activation of the PF–PC synapse; second, it could result from a synapse between the ascending portion of GC axons and the PC dendrites. The latter hypothesis is in accordance with Llinás [29], who suggested that the ascending portion of GC axons provides the major granular input into the PC. Moreover, the synapses along the ascending segments have a higher release probability [33], but the large majority of the PF–PC synapses do not generate functional links [34]. Recent studies [35] confirm the initial proposal of Llinás [29] about the vertical (patchy) organization of the PC and demonstrate the existence of two specific excitatory synapses spatially segregated on the PC dendrite. Of interest, the ascending segment synapses are made exclusively on the smallest-diameter PC dendrites and present a vertical orientation roughly perpendicular to that formed by the PF–PC dendrites (horizontal orientation) (Fig. 2 of Lu et al., [35]). According to the present results, these new elements may be interpreted as follows: (1) the earlier SS response may be explained by a shorter pathway (ascending axon to the PC dendrite shorter than ascending axon+PF pathway to PC dendrite), and the ascending axon synapse made on the smallest dendrite could facilitate the earlier recruitment of this PC response; or (2) the perpendicular orientation of the two distinct synaptic inputs may explain why the N3 but not the N2 is reversed during the same in-depth recording.

Finally, the ascending axons have been proposed to be mainly hard wired and resistant to LTD [33] and to play the role of event detectors. In contrast, the PF–PC synapses display short- and long-term synaptic plasticity [2], [8], [9], [19]. In this context, the absence of an amplitude decrease in the N2 after the 8-Hz stimulation imposed in this study might indicate support of this LFP component by the ascending axon–PC synapse while the N3 was more directly related to PF–PC synapses.

### Evidence for cerebellar LTD in alert animals

A wealth of evidence supports the notion that the LTD effect demonstrated here corresponds to the cerebellar learning theory [3]–[6], [36]. In that theory, the CF induces LTD at the PF–PC synapses, specifically for the PFs that were active at or just before the occurrence of the CF input. The present paradigm does not require any type of motor learning, and both climbing and mossy fiber inputs are involved. As in the original formulation of this theory, we demonstrated here that whisker stimulation consistently resulted in the conjunctive recruitment of both climbing and mossy fibers, as evidenced by the SS firing increase shortly followed by CS firing. Moreover, we demonstrated that during the 8-Hz stimulation, the timing of this conjunctive recruitment was conserved, and the evoked CS reached a high frequency rate. Although no motor learning task is required in the present sensory stimulation paradigm, the increase of the CS frequency during the 8-Hz stimulation period is reminiscent of the reported increase in CS during the learning period of a motor task [37]. After that pioneering work, the increase in CS frequency during behavioral learning was confirmed [38], [39]. Furthermore, the seminal *in vitro* study of Ito and Kano [2] demonstrated that conjunctive stimulation of PFs and CFs at 4 Hz induces an LTD in the field potential associated with postsynaptic excitation in dendrites of PCs and other cortical cells. Consistent with that evidence, our findings in alert animals indicated that a peripheral stimulation that activated both climbing and mossy fibers could induce LTD in the N3 component.

Llinás et al. [40] promoted an alternative to the view that LTD played a role in learning, hypothesizing that LTD may be neuroprotective against Ca^2+^-mediated excitotoxicity. In that context, the present 8-Hz stimulation may be viewed as a favorable influence on Ca^2+^ influx overflow in which a 5-fold increase in the CF inputs triggers a powerful Ca^2+^ influx [41]. In addition, the concomitant increase in PF input may contribute to a more focal Ca^2+^ increase [42], [43]. Thus, the present LTD in the N3 components might be used as an electrophysiological index of PC neuroprotection.

Worth noting, 8 Hz is the dominant frequency of whisker movements during exploration in rats [44]. In an important study about long-term plasticity in the mouse sensorimotor cortex, Mégevand et al. [45] demonstrated that passive stimulation of whiskers at 8 Hz induces plasticity of whisker-evoked cortical responses. In that relevant work, the authors produced rhythmic movement of whiskers in the anaesthetized animals for 10 min. In addition, when mice were exposed to environmental enrichment, the authors observed a lasting increase in the amplitude of whisker-evoked responses in the somatosensory cortex that occluded further potentiation.

### Relationship between PC firing and LFP

Recent work showed that both SS and CS were evoked in the same PCs in response to whisker dermatome stimulation in both alert and anesthetized mice [22], [25], [46]. The dominant SS response consisted of a short excitation followed by inhibition, as originally described in the rat [26] and confirmed in other stimulation protocols [22], [25], [46]–[48]. This SS pattern was explained by the feedforward excitation–inhibition sequences described during direct PF stimulation *in vitro* [49], [50] or *in vivo* [28]. The same peripheral stimulation produced a CS at about 20 ms in alert mice [25], [46], similar to our present observations. This evidence demonstrated that climbing and mossy fibers are simultaneously recruited by a single peripheral stimulation. The fact that the first SS response occurred very early (∼3.0 ms) and coincided with N2–N3 peak latency strongly supports the proposal that these components had a postsynaptic nature and, in the case of the N3, originated at the PF–PC synapses as the inversion and collision data suggest. The timing of the SS synchronized on the N3 field reinforced this fact. This N3 plasticity is also supported by the finding that PF–PC synapses display short- and long-term synaptic plasticity, as shown in different preparations [2], [8], [9], [19].

### Is the present LTD dependent on CF activity?

Previous work has shown that in slices, LTD is induced when PF activation precedes CF activation by 250 ms; moreover, simultaneous activation or reversal of the temporal sequence impairs LTD [51]. However, both the blockade of GABA_A_ receptors and the increase of the pairings toward 600 stimuli could induce LTD. The latter situation could have arisen in the present study because we used a total of 4,800 electrical stimuli (8 Hz for 10 min), which would give rise to about 3,180 conjunctive PF–CF activations (CF stimulation at ∼5.3 Hz for 10 min) for inducing LTD in alert animals. We also confirmed conservation of the temporal order between the climbing and parallel fiber activations during the 8-Hz stimulation. Furthermore, the SS mean frequency doubled, and the evoked CS frequency reached 5.3 Hz compared to 0.69 Hz in the control condition. This finding corresponds to the basic requirement of the classical LTD view [52]. However, we note that a strong activation of the PF alone is sufficient to produce, *in vitro*, a form of LTD that is sustained by a Ca^2+^ transient [43]; nevertheless, the existence of these forms of plasticity in alert animals remains to be shown. It is difficult to compare an LFP recorded in alert animals to an excitatory postsynaptic potential recorded in a slice; the LFP amplitude is greatly dependent on the number of synchronized neuronal entities recruited by the peripheral stimulus, as demonstrated in the somatosensory cortex of awake mice [53]. In the present study, the first PF–PC excitation, the evoked SS, occurred ∼20 ms before the evoked activation of the CF. This temporal link between PF and CF activities may indicate a role similar to that revealed in PC spines, when PF activation occurred 50–200 ms before CF activation [54] and gave rise to supralinear calcium transients.

### LTD and the timing effect

In the context of pattern recognition theory, *in vitro* and PC model studies [55] have shown that a PF input alone is sufficient to evoke, upon spontaneous SS firing, a highly reproducible pattern of three SS spikes followed by a pause. In that work, the timing of the second and third evoked spikes was delayed after LTD induction, without the contribution of any other neuronal elements in the cerebellar network. This pattern was very similar to what we observed in the present study with trigeminal input. However, we cannot rule out the possibility that the mossy fiber–GC synapses were implicated in the observed time-delay effect. The time shifts were roughly the same for the N2 and N3; thus, this temporal plasticity may have occurred in the GC layer. This alternative has been demonstrated *in vitro* [30] and in the anesthetized rat [20]; moreover, it gains theoretical support from the notion that the mossy fiber–GC synapses, regulated by Golgi cells, form a template for timing adjustments of the mossy fiber input [56].

### Do the molecular inhibitory interneurons contribute to the present LTD?

The inhibitory interneurons are excited by both PF [31] and CF inputs [19]; therefore, they might be involved in the present plasticity. The N2 and N3 latency shifts fit relatively well with the PC output spike shift measured *in vitro* after the induction of an inhibitory postsynaptic potential [50]. However, the contribution of inhibitory synapses to the evoked SS timing does not necessarily imply their participation in LTD. Indeed, the fact that the pairing protocol of CF+molecular interneuron stimulation gave rise to an inhibitory synapse LTD [50] argues against the possibility that this inhibitory synapse was responsible for the LTD observed in the present study.
